# Silane modified upconversion nanoparticles with multifunctions: imaging, therapy and hypoxia detection

**DOI:** 10.1038/srep22350

**Published:** 2016-02-29

**Authors:** Shihan Xu, Xinran Zhang, Hongwei Xu, Biao Dong, Xuesong Qu, Boting Chen, Shuang Zhang, Tianxiang Zhang, Yu Cheng, Sai Xu, Hongwei Song

**Affiliations:** 1 State Key Laboratory on Integrated Optoelectronics, College of Electronic Science and Engineering, Jilin University, 2699 Qianjin Street, Changchun, 130012, P. R. China; 2 College of Physics, Jilin University, 2699 Qianjin Street, Changchun, 130012, P. R. China; 3 Department of Physics, Changchun Normal University, Changchun 130032, P. R. China

## Abstract

Herein, we report a facile route to synthesize silane coated upconversion nanoparticles (UCNPs@silane) with an ultrathin layer (the thickness: 1–2 nm), which not only provides good biocompatibility, but also affords hydrophobic interspace to load organic molecules to realize multifunctions. Besides the function of upconversion imaging of UCNPs, cancer therapy and oxygen level detection can also be realized by the addition of chemotherapy drug, PTX, and oxygen sensitive molecules, Platinum (II) octaethylporphine (PtOEP). In bio-experiments, besides the MTT assays, therapy efficacy of UCNPs@PTX@silane can also be detected with the confocal laser scanning microscopy (CLSM) by staining methods. UCNPs@PtOEP@silane can afford minimally invasive analysis of dissolved oxygen and then respond sensitively to the variance of intracellular oxygen concentration affected by therapeutic UCNPs@PTX@silane.

In recent years, integration of multimodal treatment strategies has greatly enhanced anticancer efficacy and optimized therapy due to the synergistic or combined effects^1^,^2^,^3^. This promising approach of co-assembly of multifunctional agents attracts great attentions which is dedicated to constructing multifunctional platforms including iron oxide^4^,^5^, gold^6^,^7^,^8^, lanthanide nanocrystals^9^,^10^,^11^,^12^,^13^ (NCs) and polymers^14^,^15^,^16^,^17^. Although conceptually impressive, these researches are still at an early stage. Co-assembly of several inorganic or organic-based nanoparticles (NPs) has been proposed to integrate various functions^18^,^19^. However, each type of nanoparticles also displays distinct limitations. For instance, mesoporous silica can be integrated with gold nanorods^20^,^21^,^22^ or upconversion NCs^23^,^24^,^25^ to display therapeutic effects and imaging functions. Though the promising functions can be obtained, the size of the composites increases greatly, which may cause the limitations on the broad clinical application because of concerns about their long-term safety and poor targeting properties *in vivo*, since the nanoparticles with larger size can be cleared rapidly by liver and spleen^26^. Another example is about the “soft” organic nanoparticles, such as paclitaxel (PTX)-loaded polymeric micelles^27^,^28^,^29^. They are more approached to clinical trials for the treatment of human cancers due to the excellent biocompatibility. However, besides the size problem (normally large size), this kind of composite always lacks the imaging function.

Complexity of fabrication also remains on many multifunctional nanoparticles since the poor biocompatibility always follows the nanoplatforms preparation. The great conflict between the size and multifunction of the composite becomes the major concern on the integration of various clinically relevant imaging functionality and therapeutic property. Therefore, the multifunctional nanoplatform needs to be more biocompatible with small size^29^ and “smart” enough to overcome the biological barriers caused by larger size. Recently, we developed a facile strategy for multifunctional platform designed by amphiphilic silane modification with ultrathin thickness (1 nm) at room temperature (RT)^30^. In this strategy, the coating layers can not only convert hydrophobic inorganic nanoparticles into hydrophilic ones, but also afford the place for loading organic molecules for multifunctional bio-applications, without increasing size. In this work, this strategy was further applied in cancer therapy and cell viability detection (by measuring the oxygen level) in real time by adding the anticancer drug, PTX, and oxygen sensing molecules, Platinum (II) octaethylporphine (PtOEP) into this structure. As shown in Fig. 1, NaYF_4_:Yb^3+^, Er^3+^ upconversion nanoparticles (UCNPs) with small size serve as an inorganic core, then they were encapsulated with silane by hydrophobic interactions and an ultrathin layer (1–2 nm) providing water solubility and luminescent stability evolved in this hydrolysis process. The organic molecules were loaded into the ultrathin hydrophobic interspaces between the UCNPs and silane via hydrophobic interactions. In this way, upconversion imaging, chemotherapy, and oxygen sensing functions can be achieved within ultrasmall nanoparticles.

It is worth noting that, intracellular oxygen concentration is lower than that of extracellular space due to the respiratory activity for adenosine triphosphate (ATP) production. Hypoxia is more remarkable for tumor cells due to the imbalance between the cancer cell proliferation and limited oxygen supply^31^. From this viewpoint, we intend to monitor the apoptosis process based on minimally invasive of UCNPs@PtOEP@silane. Due to this novel structure, the cell imaging, therapy and oxygen sensing can be monitored in real time under confocal laser scanning microscopy (CLSM).

## Results and Discussion

### Synthesis of UCNPs@PTX/PtOEP@silane

In this work, both hydrophobic molecules PtOEP and PTX can be loaded into the interspace between the UCNPs and silane due to the hydrophobic interaction. Though the two organic molecules can be loaded inside together, it is difficult to optimize the two different functions due to the limited space in the ultrathin interspace.

**Figure 1:**
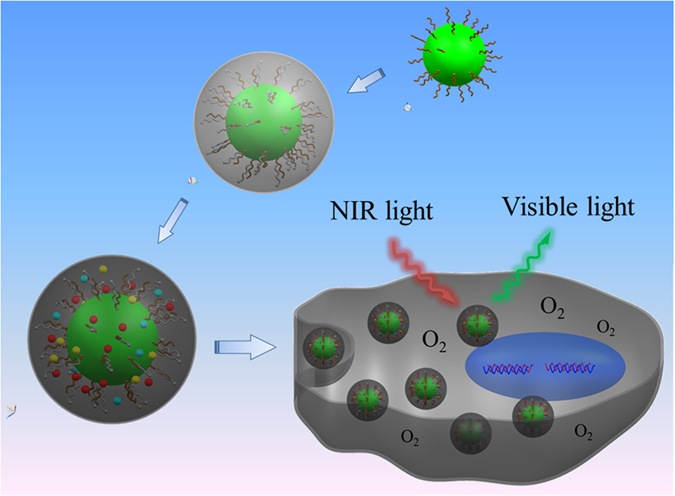
Schematic illustrations of the synthesis process of UCNPs@silane and UCNPs@PtOEP/PTX@silane. The PTX and PtOEP molecules were encapsulated within the ultrathin coating layer (1–2 nm). After these composite NPs were internalized by tumor cells, the multifunctional NPs could emit visible light under NIR irradiation for upconversion imaging. In addition, PTX and PtOEP can play the function of therapy and oxygen sensing. The colorful dots mean different kinds of hydrophobic molecules, such as PTX and PtOEP.

**Figure 2:**
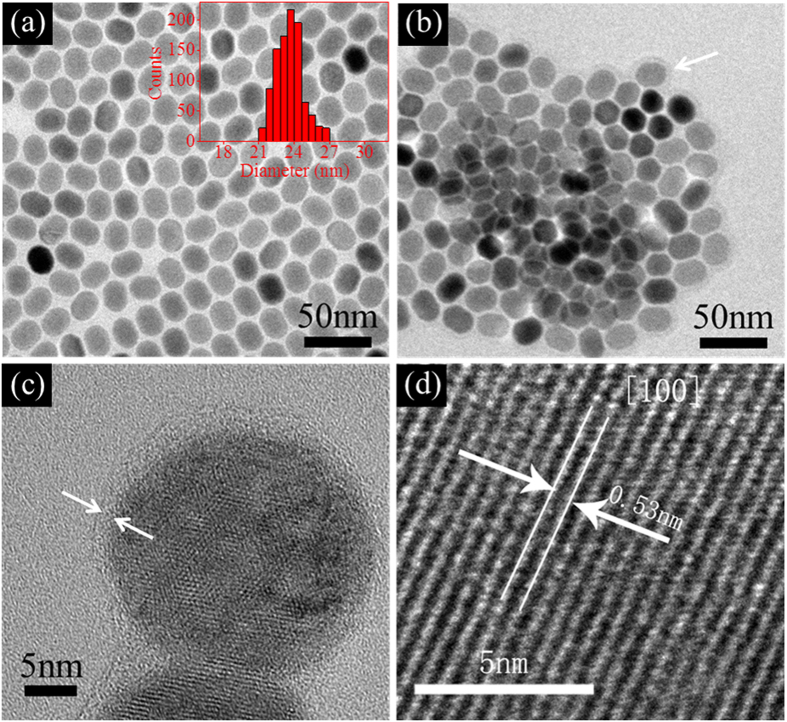
The TEM images of (a) NaYF_4_:Yb^3+^, Er^3+^ UCNPs and (b) UCNPs@PTX/PtOEP@silane, the inset of (a) shows the size distribution of the UCNPs. (**c**) HRTEM image of UCNPs@PtOEP/PtOEP@silane, and the thin layer on the surface of NaYF_4_:Yb^3+^, Er^3+^ UCNPs is 1–2 nm. (**d**) The lattice fringes belonging to NaYF_4_:Yb^3+^, Er^3+^ UCNPs.

The facile and straightforward fabrication procedure of UCNPs@PTX@silane and UCNPs@PtOEP@silane based on a one-step self-assembly approach is schematically illustrated in Fig. 1. NaYF_4_:Yb^3+^, Er^3+^ UCNPs were synthesized by a solvothermal method according to the previous report^32^. The UCNPs are spherical with an average size of 23 nm (Fig. 2a), showing clear lattice fringes with 0.53 nm lattice spacing in accordance with (100) planes of hexagonal NaYF_4_ (Fig. 2d). Further evidence for the hexagonal NaYF_4_ crystals is given by the XRD pattern (JCPDS 28-1192) in Supplementary Fig. S1. After surface modification, the NaYF_4_:Yb^3+^, Er^3+^ UCNPs and amphiphilic silane with 18C alkyl chain are self-assembled to form spherical NPs with a coating layer about 1 nm (Fig. 2b,c). The dehydration and shrinkage of the nanoparticles during the process for TEM observation^33^ might lead to the aggregation of nanoparticles.^34^ Amphiphilic silane is used here to encapsulate hydrophobic cores (UCNPs) via the hydrophobic interactions between the alkyl chains of silane and the octadecyl group of oleic acid on the surface of UCNPs. Silane with different lengths of alkyl chains, were selected, while they own similar coating thickness (see Supplementary Fig. S2). It indicates that the hydrophobic interactions can make relative soft coating layers with a similar thickness^30^. The FTIR spectra (see Supplementary Fig. S3) confirm the occurrence of hydrolysis and poly-condensation reaction. Furthermore, some hydrophobic molecules (PTX and PtOEP) could be loaded into the interspace between cores and silane. It needs to be mentioned that the hydrophobic core, NaYF_4_:Yb^3+^, Er^3+^ UCNPs, is necessary for this modification strategy, and if there is no UCNP inside, the hydrophobic molecules, like PTX would be in the form of white precipitation, instead of normal transparent colloids (the left cuvette in Supplementary Fig. S4b). As to the case of UCNPs@PtOEP@silane, the color of colloids changed to weak pink (see the right one in Supplementary Fig. S4b), indicating the successful loading of PtOEP. Pt element besides Yb and Er (the elements Na, F and Y are not showed in this figure), can be detected by Energy-dispersive X-ray (EDX) analysis (see Supplementary Fig. S4a). In addition, they can exhibit remarkable stability without any leakage for 4 month and the zeta potentials of residual UCNPs@PtOEP@silane and UCNPs@PTX@silane samples are about −21.0 mV and −21.6 mV (see Supplementary Fig. S5) measured by dynamic light scattering (DLS), which proves their stability in water^34^,^35^.

### Upconversion imaging

When excited by the 980 nm laser, the UCNPs@silane colloids with different lengths of alkyl chains showed similar visible emissions spectra, peaked at 420, 528, 540, and 660 nm, corresponding to the transitions from ^2^H_9/2_, ^2^H_11/2_, ^4^S_3/2_, and ^4^F_9/2_ excited states to the ^4^I_15/2_ ground state of Er^3+^ (see Supplementary Fig. S6). Digital pictures in the inset show the bright green emission of the UCNPs colloids under 980 nm laser excitation, convincing the good water solubility after the successful modification of UCNPs.

**Figure 3:**
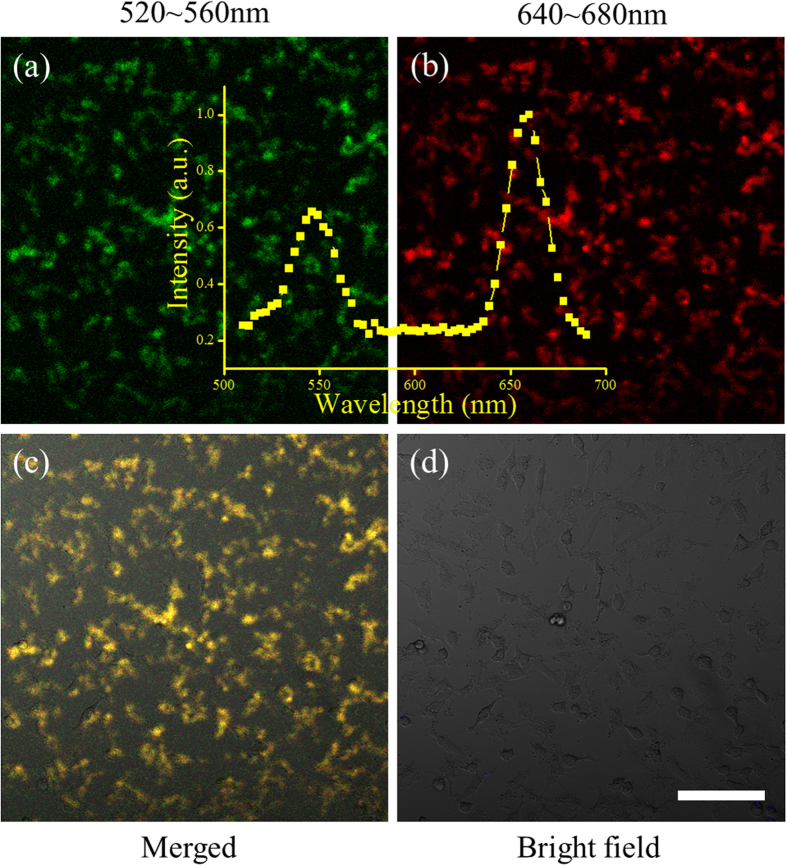
CLSM images of A549 cells treated with UCNPs@silane when irradiated by 980 nm laser, and the green channel (a: 540 ± 20 nm) and red channel (b: 660 ± 20 nm) images are corresponding to the green and red light emissions of UCNPs. The inset is the upconversion emission spectrum monitored by CLSM with the step of 3 nm and the bandwidth of 15 nm. (**c**) is the merged image of (**a**) and (**b**), and (**d**) is the bright field image. The scale bar is 100 μm.

In order to verify the up-conversion imaging properties of UCNPs@silane under 980 nm laser excitation, CLSM was carried out on A549 cells incubated with UCNPs@silane (100 μg/mL) for 24 h. The green and red upconversion emission of UCNPs are monitored with green channel set from 520 to 560 nm and red channel set from 640 to 680 nm, respectively (Fig. 3a,b). The UCL spectrum detected in the region of interest (ROI) is in good agreement with that detected by spectrometer, confirming the bioimaging properties of UCNPs@silane. The merged image and bright field image (Fig. 3c,d) show that the cells maintained normal morphology, indicating a good biocompatibility of UCNPs@silane.

### The optimal amount of PTX

We have previously reported a kind of nanocomposites based on NaYF_4_:Yb^3+^, Er^3+^ UCNPs for efficient encapsulation of Eu complex^30^. We found that the silane with longer alkyl chains, such as 18C, can contain more molecules than those with shorter chains. In this work, PTX loading efficiency was measured with UCNPs modified by 18C silane. The weight ratios of PTX to NaYF_4_:Yb^3+^, Er^3+^ UCNPs were adjusted to 0.5:5, 0.6:5, 0.7:5, 0.8:5, and 0.9:5, in the meantime, the amount of UCNPs was fixed to 5 mg. PTX could be totally encapsulated with the ratio of 0.5:5 and 0.6:5, which are corresponding to an encapsulation efficiency of 100% and the drug loading efficiency was 3.85% and 4.62%, respectively. When formulated at 0.7:5, there would appear white precipitation in the dialysis membranes. As the amount of PTX further increases, the precipitation grows. For the loading efficiency, it can be calculated by measuring the amount of PTX precipitation. According to the standard curve of the absorption of PTX at 227 nm (see Supplementary Fig. S7a) and precipitating PTX (see Supplementary Fig. S7b), the amount of loading PTX can be calculated to be 673, 738.5 and 789.5 μg, corresponding to the encapsulation efficiency of 96.1%, 92.3% and 87.7% for the weight ratio of 0.7:5, 0.8:5, and 0.9:5, respectively. And the drug loading efficiency is 5.1%, 5.58% and 5.94%, respectively. Though the loading amount increases, the encapsulation efficiency drops. The corresponding EE, DLE, as well as the PDI value from DLS measurements are listed (see Supplementary Table S1). As the encapsulation efficiency decreases below 100%, the loading amount of PTX increases, but the dispersity becomes worse. It can be confirmed by the minimum PDI value, which is 0.193 at the weight ratio of 0.6:5.

### Therapeutic efficiency of UCNPs@PTX@silane *in vitro*

**Figure 4:**
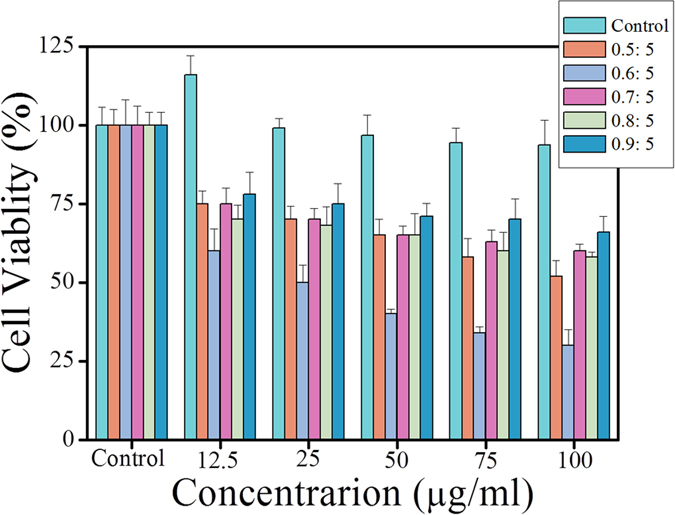
Relative cell viability when incubated with different concentrations of UCNPs@PTX@silane (different weight ratios of PTX to NaYF_4_:Yb^3+^, Er^3+^ UCNPs) for 24 h. The experiments were repeated three times, all with similar results. The data are presented as the mean ± SD (for each group, n = 3).

To investigate the therapy efficiency of the formulated NPs, we conducted the MTT assays on both UCNPs@silane and UCNPs@PTX@silane. As shown in Fig. 4, UCNPs@silane almost has no side-effect on cells, even under high concentration of 100 μg/ml, and the viability of cells is still more than 93%. When the concentration further increases, it would lead to cell apoptosis at a great extent and irregular results. Furthermore, under low concentration the cells appear proliferation at some extent^36^, proving this modification method is beneficial for biological applications. In contrast, because of containing PTX, which could interfere with mitotic spindle function by inducing tubulin polymerization and ultimately arrest cells in the G2/M phase of mitosis^33^, UCNPs@PTX@silane shows significant cytotoxicity on A549 cells by the MTT assays. The cell viability decreases as the concentration of UCNPs@PTX@silane grows. With the same concentration of NPs, the samples with different weight ratios of PTX to NaYF_4_:Yb^3+^, Er^3+^ UCNPs also have different results by the MTT assays. The sample (the weight ratio of PTX to UCNPs is 0.6:5) shows the highest cytotoxicity in the concentration of 100 μg/ml, and only 30% living cells exist. The results also convince that 0.6:5 is the optimized ratio for therapeutic efficiency.

**Figure 5:**
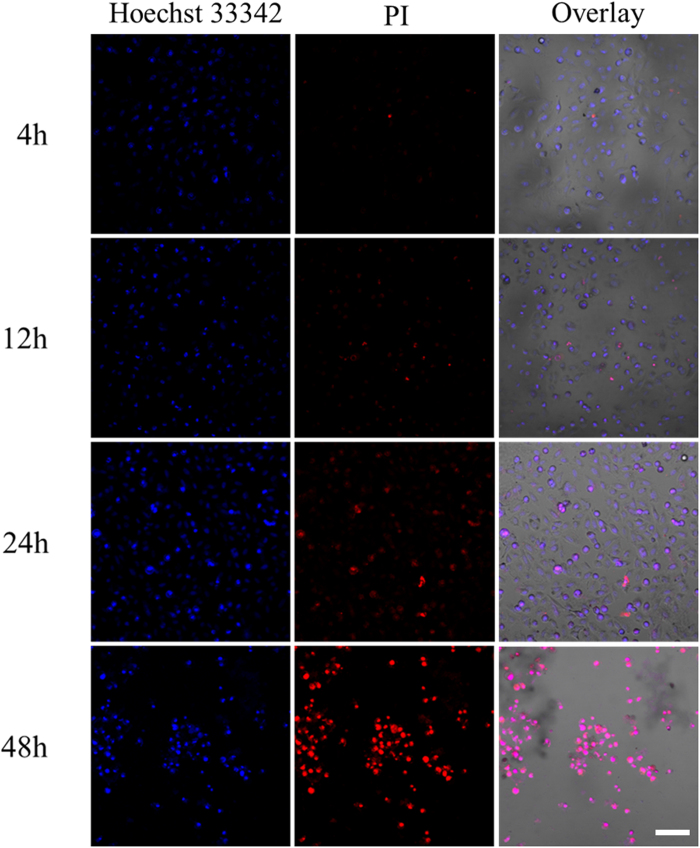
CLSM images of A549 cells treated with UCNPs@PTX@silane (100 μg/ml). Fluorescence images were measured between 425 and 475 nm for Hoechst 33342 (λex: 405 nm), and between 600 and 660 nm for PI (λex: 488 nm). The images are fake color. Cell nucleus stained by Hoechst 33342 is shown in blue, and dead cells stained by PI are shown in red color. The scale bar is 100 μm.

The therapeutic effect was further investigated by CLSM. After different incubation time with UCNPs@PTX@silane (the weight ratio of PTX to UCNPs is 0.6:5), the dishes were washed with PBS for three times to remove the extra UCNPs@PTX@silane. Then, Hoechst 33342 which can effectively combine with DNA and propidium iodide (PI) which could only be taken-up by necrotic cells with broken membranes were added to the dish to monitor the cell position and its viability, respectively. CLSM with the multichannel function was used to detect the blue fluorescence of Hoechst 33342 and red fluorescence of PI under the excitation of 405 nm and 488 nm lasers. Figure 5 shows the confocal images when A549 cells were incubated with UCNPs@PTX@silane for various time intervals, 4, 12, 24 and 48 h. The rows display the emission of Hoechst 33342, PI and the merged image, respectively. The images stained by Hoechst 33342 show the configuration of nucleus inside cells. From the PI stained images, as the incubation time increases, “bright red” points appear more and more, which indicates the death of cells induced by PTX is increasingly severe. After 24 h, most cells appear red, which is in accordance with the result of the MTT assays. Then 48 h, all cells seem red, indicating that no living cell exists.

### Optical properties of UCNPs@PtOEP@silane

**Figure 6:**
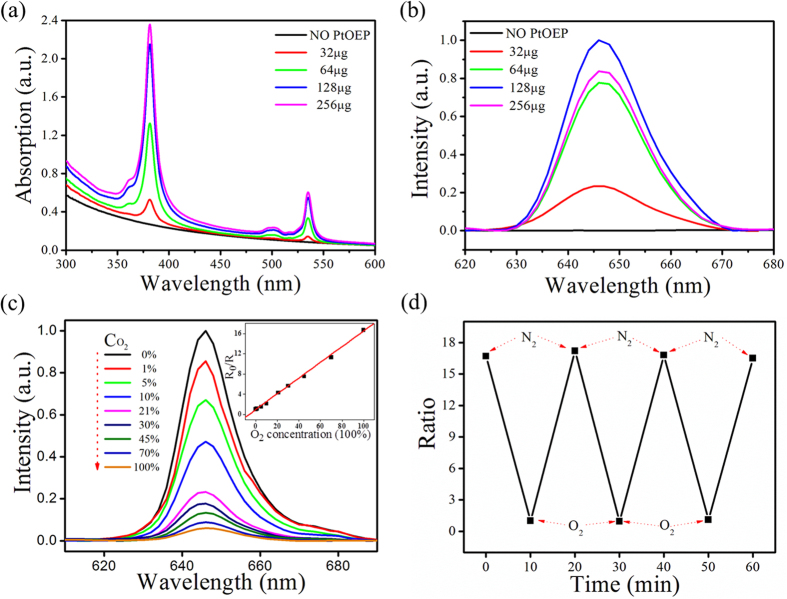
(**a**) The absorption spectra of UCNPs@PtOEP@silane with different amounts of PtOEP. (**b**) The emission spectra of UCNPs@PtOEP@silane with different amounts of PtOEP (under 532 nm excitation). (**c**) Response of UCNPs@PtOEP@silane towards various concentrations of oxygen at room temperature. The inset is the Stern-Volmer plot. (**d**) Reversibility of UCNPs@PtOEP@silane responding to dissolved oxygen. The fluorescence ratios are plotted versus the experimental time.

Luminescence sensing and imaging is a widely used technique, especially in biological field^37^. PtOEP, a hydrophobic oxygen sensor, is chosen here to sense oxygen. Photophysical analysis of UCNPs@PtOEP@silane reveals that it has a characteristic red emission from 630 nm to 670 nm and two absorption bands peaked at 380 nm and 540 nm (Fig. 6a,b). The emission and absorption intensities grow in accordance with the augment of PtOEP and obtain maxima as the amount of PtOEP is 128 μg. Quenching of fluorescence happens as the amount of PtOEP further increases. Oxygen quenching ability of UCNPs@PtOEP@silane (the containing PtOEP is 128 μg) was quantitatively analyzed in an airtight cuvette. Figure 6c shows emission spectra of UCNPs@PtOEP@silane at various O_2_ concentrations. The emission intensity valued at 645 nm is highly sensitive to O_2_, showing a 15.7 fold increase from oxygen saturated to oxygen free condition, demonstrating that UCNPs@PtOEP@silane can serve as an effective agent for hypoxia detection. The relationship between the concentration of oxygen and the emission intensity can be depicted as a Stern-Volmer plot in the inset of Fig. 6c, and the equation is showed as below,


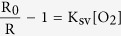


where Ksv is the Stern–Volmer constant and [O_2_] represents the concentration of oxygen. From the linear relation between R_0_/R and [O_2_], the sensitivity can be calculated to be 16.7, which is high enough to distinguish hypoxic conditions. Furthermore, UCNPs@PtOEP@silane also shows remarkable reversibility and obtains a complete recovery in each time, as depicted in Fig. 6d.

### Sensing intracellular oxygen

**Figure 7:**
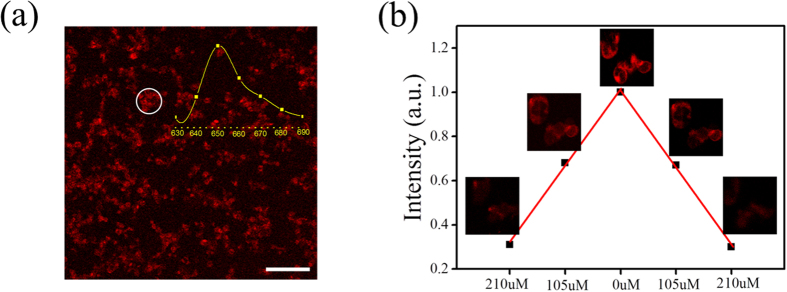
(**a**) The confocal fluorescence image detected at 650 nm with the bandwidth of 15 nm. Inset: the emission spectrum of ROI (the white circle area) was also monitored from 630 to 690 nm with step of 10 nm and bandwidth of 15 nm. (**b**) The emission intensity of ROI changes as the concentration of oxygen changes. The scale bar is 100 μm.

Before bio-experiments, the cytotoxicity of UCNPs@PtOEP@silane was measured by the MTT assays and the results indicate that the cells remain high viability, even with high concentration of 100 μg/ml (Supplementary Fig. S8). The emission spectrum of UCNPs@PtOEP@silane (in the inset of Fig. 7a) was recorded by focusing on the ROI by CLSM (see details in Supplementary Fig. S9), which was in accord with the spectrum monitored by spectrometer shown in Fig. 6b. This result also confirmed the well dispersion of UCNPs@PtOEP@silane inside A549 cells.

To investigate the hypoxic response behavior of A549 cells, they were incubated with UCNPs@PtOEP@silane (100 μg/ml) for 24 h. Before measuring oxygen sensing ability *in vitro*, the pure air was pumped into the sealed confocal dish for 30 min to make an equilibrium condition. Then the confocal images were captured with the emission band set between 640 nm and 660 nm under the 405 nm laser excitation. Quite weak emission signal was detected as the maximal oxygen concentration (210 μM) was reached shown in Fig. 7b. Then the mixed gas (N_2_ 90%, O_2_ 10%) and pure N_2_ were pumped into the dish for 30 min, respectively. The change of signal intensity was positively correlating to the O_2_ concentration. Strong red emission of the UCNPs@PtOEP@silane was accompanied by the N_2_ equilibrium condition. Note that, as the nitrogen replacing air, there was a 2.3-fold increase for the emission intensity, while it behaved a 4.3-fold increase in an airtight cuvette. As the intracellular circumstances around UCNPs@PtOEP@silane were very complicated and the oxygen concentration differed among the subcellular organelles, this phenomenon occurred. Therefore, intracellular oxygen quenching was not as effective as in water. As the concentration of oxygen increased, the emission intensity of PtOEP droped and this dynamic process convinced the oxygen sensing capability of UCNPs@PtOEP@silane *in vitro*.

### Intracellular oxygen concentration changes caused by therapeutic effects of UCNPs@PTX@silane

After A549 cells were incubated with UCNPs@PtOEP@silane for 24 h and washed with PBS for three times, the fresh medium with UCNPs@PTX@silane (100 μg/ml) was added into the dish. The sealed dish was put under the CLSM with heating units which ensured the physiologic temperature in the dynamic process. UCNPs@PTX@silane was proved to behave high therapy efficacy.

**Figure 8:**
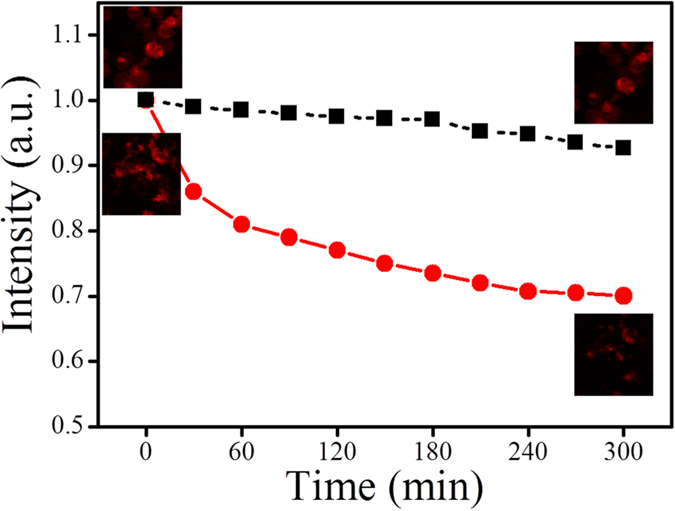
Red plots: the normalized emission intensity of UCNPs@PtOEP@silane was plotted accompanied with experimental time, after adding UCNPs@PTX@silane into the dish. Black plots: the normalized emission intensity of UCNPs@PtOEP@silane after adding UCNPs@silane. The insets are the corresponding interest areas.

After adding UCNPs@PTX@silane, the average emission intensity of ROI was monitored as shown in Fig. 8. The confocal images were caught at the interval of 30 min with the emission bandwidth set between 640 and 660 nm. In this whole therapeutic process of 5 h, the red line corresponded to the theronostic group, and its average emission intensity decreased very fast at first and then slowly, which was positively correlated with the cellular consumption of oxygen. In this process, emission intensity of PtOEP decreased about 30%. It can be explained that the PTX influenced the function of subcellular organelles, including mitochondria, so the oxygen consumption activity decreased due to the abnormal situation, though the tumor cells were not really “dead” in the early stage. In contrast, the average emission intensity of control group only showed less than 5% decrease, which may be caused by the irradiation of laser.

These results indicated that intracellular oxygen quenching happened and can be detected by the emission intensity change of UCNPs@PtOEP@silane, in addition, the interrelated change can be used to evaluate the cell viability.

## Conclusion

In summary, we reported a facile method to synthesize UCNPs@silane with an ultrathin layer thickness of 1–2 nm, which can afford a hydrophobic space to hold hydrophobic molecules to realize multifunctions. By encapsulation PTX and PtOEP in the interspace between UCNPs and silane, upconversion imaging, chemotherapy, and oxygen sensing can be achieved within the ultrasmall nanoparticles. The cytotoxicity assay demonstrated good biocompatibility and low toxicity of UCNPs@silane, while UCNPs@PTX@silane showed great therapy efficacy to A549 cells, which was further detected with a CLSM system by monitoring the cell position and viability in real time. For the early stage of the therapy process, UCNPs@PtOEP@silane can afford invasive cellular oxygen analysis and respond sensitively to the hypoxia of tumor cells. The qualitative analysis can illustrate the viability of the tumor cells after incubated together with UCNPs@PTX@silane. We are actively pursuing more sensitive and responsive NPs with our new structure design, for tumor cell ablation and fluorescence imaging of intracellular oxygen within subcellular organelles, to better understand the specific physiological and pathological process of tumor cells when the therapeutic effects occur.

## Methods

### Materials

YCl_3_·6H_2_O (99.999%), YbCl_3_·6H_2_O (99.999%) and ErCl_3_·6H_2_O (99.999%) were supplied by Inner Mongolia Chemical Reagent Company. Octadecene (ODE), oleic acid (OA), hexyltrimethoxysilane (6C silane), octyltrimethoxysilane (8C silane), dodecyltrimethoxysilane (12C silane) and trimethoxy(octadecyl)silane (18C silane) were bought from Sigma-Aldrich. NaOH, NH_4_F, tetrahydrofuran (THF) were obtained from Alfa Aesar. PTX and 3-(4, 5-dimethylthiazol-2-yl)-2, 5-diphenyltetrazolium bromide (MTT) were purchased from Sigma-Aldrich. All chemicals were used without further purification. Doubly distilled water was used in all experiments.

### Synthesis of NaYF_4_:Yb^3+^ (20%), Er^3+^ (2%) UCNPs

Hexagonal-phase NaYF_4_:20%Yb^3+^, 2%Er^3+^ UCNPs were synthesized following a recipe as below. YCl_3_·6H_2_O (0.78 mmol), YbCl_3_·6H_2_O (0.20 mmol) and ErCl_3_·6H_2_O (0.02 mmol) were mixed together in a three-necked flask. 6 mL oleic acid and 15 mL ODE were also added in the flask. Then this solution was heated to 160 °C and kept in a constant temperature condition with continuously stirring under the protection of nitrogen. After 1 h, the solution became homogeneous, and then was cooled down to room temperature. The as-prepared solution containing NaOH (2.5 mmol), NH_4_F (4 mmol) and methanol (5 mL), was added dropwise to the three-necked flask and the resolution was further stirred for 30 min. Next, the temperature of mixed solution was increased to 125 °C and kept for 20 min. At this moment, methanol could be removed from the resolution. After removing methanol, a condenser was used to save resolution not to evaporate out, as the mixed solution would be heated to 320 °C and maintained for 1 h. After that, the solution was cooled down to 30 °C, and the resultant mixture was centrifuged at a speed of 9000 r/min and washed with ethanol/ hexane (1 : 1 v/v) three times, and the precipitation was collected for further experiments.

### Modifying NaYF_4_:Yb^3+^, Er^3+^ UCNPs by amphiphilic silane: UCNPs@silane

The NaYF_4_:Yb^3+^, Er^3+^ UCNPs and silane (6C, 8C, 12C and 18C) were mixed in THF under sonication with a 2 : 3 weight ratio and at a total concentration of 3667 ppm. After 10 minutes, 3 ml resolution was rapidly injected to 15 ml water (pH ≈ 9, adjusted by addition of ammonium hydroxide) in a conical flask. Then the conical flask was put in a water bath at 30 °C for 4 hours, and the resolution was dialyzed for 24 h at room temperature.

### Loading hydrophobic molecules in this structure

This process was same with the above one as synthesizing UCNPs@silane. The NaYF_4_:Yb^3+^, Er^3+^ UCNPs, PTX/PtOEP and silane were mixed in THF under sonication with a fixed ratio. Then the process was same as the above.

### Encapsulation efficiency (EE) and drug loading efficiency (DLE)

The anticancer drug, PTX, with various concentrations (4, 8, 16, 32, 64 μg/ml) were prepared in advance, and then their UV−vis absorbance spectra were monitored. As the absorption peaks at 227 nm is the characteristic absorbance peak of PTX, the standard curve describing the relationship between absorbance intensity and concentration of PTX was plotted. As the weight ratio of PTX to NaYF_4_:Yb^3+^, Er^3+^ UCNPs was enhanced to 0.7:5, 0.8:5 and 0.9:5, white precipitation of PTX would appear. After being dried, the precipitation was resolved in ethanol (5 ml) and the absorbance intensity was compared with the standard curve. Then the amount of PTX in precipitation can be calculated. So did the EE and DLE, according to the equations (1) and (2) showed as below:


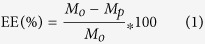



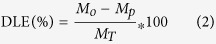


where M_0_ is the total amount of PTX initially added, M_P_ is the amount of PTX in precipitation, and M_T_ is the total amount of the UCNPs@PTX@silane.

### Characterization

Transmission electron microscopy (TEM) was carried out by using a Hitachi H-800 transmission electron microscope with an acceleration voltage of 200 kV. The crystalline structure of NPs was detected by X-ray diffraction (XRD) (Rigaku D/max-rA power diffractometer using Cu KR radiation (λ) 1.54178 Å). A Shimadzu UV-3101PC UV-Vis scanning spectrophotometer (ranging from 200 to 1100 nm) was utilized to record ultraviolet-visible (UV-Vis) absorption spectra. Fourier-transform infrared (FTIR) absorption spectra were measured on a Shimadzu DT-40 model 883 IR spectrophotometer. The pellets were prepared by adding the sample powder (0.25 mg) to KBr (25 mg). The powders were mixed homogeneously and compressed at a pressure of 10 kPa to form transparent pellets. For UCL and DCL steady-state spectra investigation, a 980 nm continuous diode laser was used to pump the samples. Dynamic light scattering (DLS, Malvern Zetasizer NanoZS) was used to investigate the size distribution and the PDI (Polydispersity index) of NPs. The emission spectra of UCNPs@PtOEP@silane were recorded with a SENS-9000 spectrometer.

### Cell culture

A549 cells were purchased from Shanghai Institute for Biological sciences, Chinese Academy of Science. The culture medium contains RPMI 1640 (GIBCO) and fetal bovine serum, and the ratio was 9 :1. A549 Cells were cultured at 37 °C with CO_2_ (5%). Trypsin (EDTA 0.02%) was used to re-suspend cells before plating.

### The cell viability assay

3-(4, 5-dimethylthiazol-2-yl)-2, 5-diphenyltetrazolium bromide (MTT) reduction assays were used to assess cytotoxicity of NPs *in vitro*. The cells were seeded onto 96-well plates at a density of 5*10^3^/well. After 24 h, the cells were attached to the plates tightly, and then incubated with different concentrations of 18C UCNPs@PTX@silane in 5% CO_2_ at 37 °C for 24 h. At the end of the incubation, MTT solution (10 μL, diluted in PBS with a final concentration of 1 mg/mL) was added, and then the cells were incubated for another 4 h. After that, the culture medium in 96 wells was discarded through using a micro-syringe and adding dimethylsulfoxide (DMSO, 150 mL) to each well. After the color of each well became stable, the absorbance was determined by using a microplate reader (BioTek, ELx800). A series wells without addition of NPs were regarded as a blank control. The cytotoxicity was expressed as the percentage of cell viability compared with the blank control.

### Confocal imaging about therapeutic effect

An Olympus FV1000 CLSM was employed to observe cells. At first, A549 cells at 1 × 10^5^ cells/well were seeded to 35 mm confocal dishes and incubated for 24 h, and then they were treated with UCNPs@silane and UCNPs@PTX@silane in fresh medium. For the cells incubated with UCNPs@silane, the upconversion luminescence imaging was carried out. A 980 nm laser under the power density of 600 mW was used to excite NaYF_4_:Yb^3+^, Er^3+^ UCNPs, with green channel monitoring the scale from 520 to 560 nm and red channel monitoring the scale from 640 to 680 nm. For those added with UCNPs@PTX@silane, incubated for different time intervals, 4, 12, 24 and 48 h, cells were stained with Hoechst 33342 and PI for 10 min, washed with PBS for three times to remove the extra dye, and then imaged by a confocal fluorescence microscope. The 405 and 488 nm lasers with the power density of 2.5 mW were used with the detection scale from 425 to 475 nm for Hoechst 33342 and 600 to 660 nm for PI.

### Stern-Volmer Plot

Different ratios of N_2_ and O_2_ were gained by a gas mixer (Wittgas, type KM60-2, Germany). The UCNPs@PtOEP@silane colloids were put in an airtight cuvette with two holes in the top, whose diameter was around 0.3 mm. Then the gas with different concentration of oxygen was pumped into the cuvette through a needle. After 30 min, the emission spectra of PtOEP under various oxygen ratio circumstances were recorded under the 532 nm excitation. Then the Stern-Volmer plot was made which indicated how emission intensity of PtOEP changed as the concentration of oxygen changed.

### Sensing oxygen under confocal laser scanning microscope

The sealed confocal dish was bubbled with nitrogen gas containing oxygen (0, 10 and 21%) for at least 10 min until the equilibration of ambient barometric pressure accomplished. Then the emission intensity of an interest area was calculated, and the relationship between oxygen concentration and emission intensity was plotted.

### The effect of UCNPs@PTX@silane on the intracellular oxygen concentration

At first, A549 cells were cultured in confocal dishes for 24 h, and UCNPs@PtOEP@silane (100 μg/ml) was incubated with cells for another 24 h. Then washed with PBS for three times, and added with fresh medium containing UCNPs@PTX@silane (100 μg/ml). The confocal images were caught at the interval of 30 min. The emission intensity of region of interest was chosen to observe how the emission intensity changed as the therapeutic process occurred.

## Additional Information

**How to cite this article**: Xu, S. *et al*. Silane modified upconversion nanoparticles with multifunctions: imaging, therapy and hypoxia detection. *Sci. Rep.* **6**, 22350; doi: 10.1038/srep22350 (2016).

## Supplementary Material

Supplementary Information
