# Supplementary material for: Silane modified upconversion nanoparticles with multifunctions: imaging, therapy and hypoxia detection
Source: Sci Rep. 2016 Feb 29;6:22350. doi: 10.1038/srep22350 (PMC4770318; doi:10.1038/srep22350)
Supplement: Supplementary Information [file srep22350-s1.pdf]

SUPPORTING INFORMATION

Silane modified upconversion nanoparticles with multifunctions: imaging, therapy and hypoxia detection

Shihan Xu[1], Xinran Zhang[1], Hongwei Xu[1], Biao Dong[1*], Xuesong Qu[3], Boting Chen[1], Shuang Zhang[1], Tianxiang Zhang[2], Yu Cheng[1], Sai Xu[1], Hongwei Song[1*]

[1]*State Key Laboratory on Integrated Optoelectronics, College of Electronic Science and Engineering, Jilin University, 2699 Qianjin Street, Changchun, 130012, P. R. China*

[2]*College of Physics, Jilin University, 2699 Qianjin Street, Changchun, 130012, P. R. China*

[3]*Department of Physics, Changchun Normal University, Changchun 130032, P. R. China*

*Authors to whom correspondence should be addressed. Email addresses: dongb@jlu.edu.cn (Biao Dong) and songhw@jlu.edu.cn (Hongwei Song).

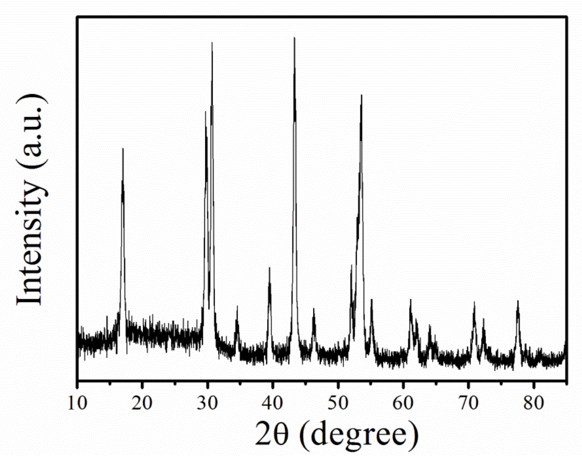

Supplementary Figure S1. XRD of hexagonal phase $NaYF_4$:Yb^{3+}, Er^{3+} UCNPs.

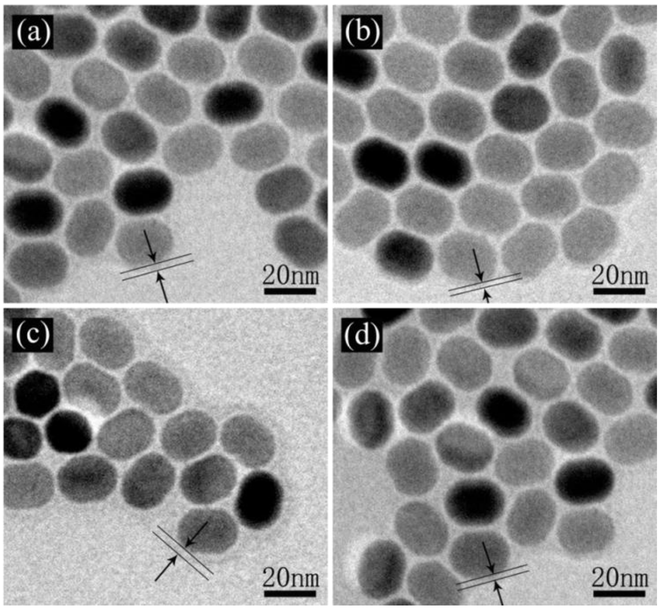

Supplementary Figure S2. TEM images of (a) 6C, (b) 8C, (c) 12C and (d) 18C silane modified UCNPs. (Silane owing different length of alkyl chains was chosen to modify the hydrophobic $NaYF_4$:Yb^{3+}, Er^{3+} UCNPs. A thin layer could be seen on the surface of $NaYF_4$:Yb^{3+}, Er^{3+} UCNPs as the black arrows point out). Different NPs show similar thickness, because hydrophobic interactions can only make the relative soft coating layer with a similar thickness.

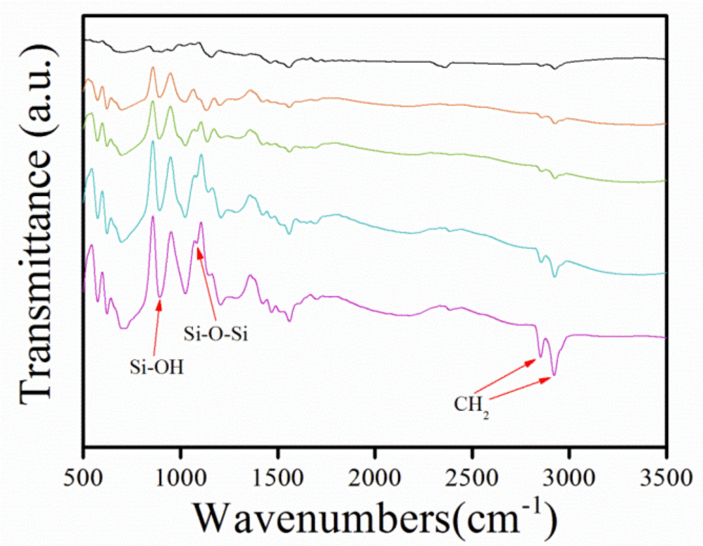

Supplementary Figure S3. The FTIR spectra of the unmodified $NaYF_4$:Yb^{3+}, Er^{3+} and silane modified $NaYF_4$:Yb^{3+}, Er^{3+} UCNPs. (From the above to below is unmodified, 6C, 8C, 12C and 18C silane modified UCNPs)

FTIR characterizations of different silane modified small NPs are shown in Figure S3, and the absorption bands at 2920 and 2850 cm^{-1} derive from the asymmetric and symmetric stretching vibrations of CH_2 and the peak intensity grows, as the alkyl chains of silane become longer. Compared with the unmodified UCNPs, there are two absorption bands appearing, 890 and 1090 cm^{-1}, which correspond to Si-OH and Si-O-Si, respectively. It further confirms that hydrolysis and polycondensation reaction occurs.

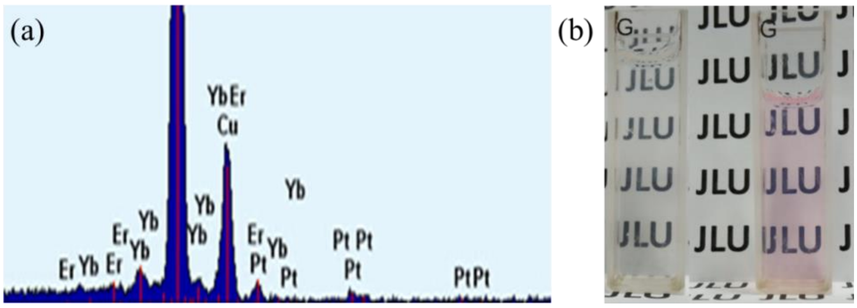

Supplementary Figure S4. (a) EDX analysis of UCNPs@PtOEP@silane. Pt, Yb and Er elements appear together. (b) Digital pictures of UCNPs@PTX@silane (left) and UCNPs@PtOEP@silane (right) are displayed. The color of weak pink proves the successful modification of PtOEP, and the left transparent resolution shows the successful loading of PTX.

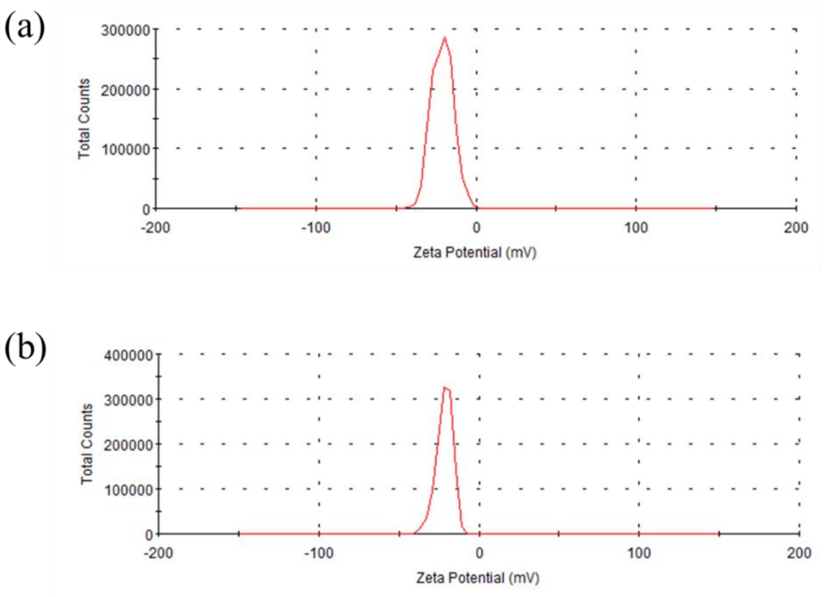

Supplementary Figure S5. The zeta potentials of residual samples: (a) UCNPs@PTX@silane and (b) UCNPs@PtOEP@silane.

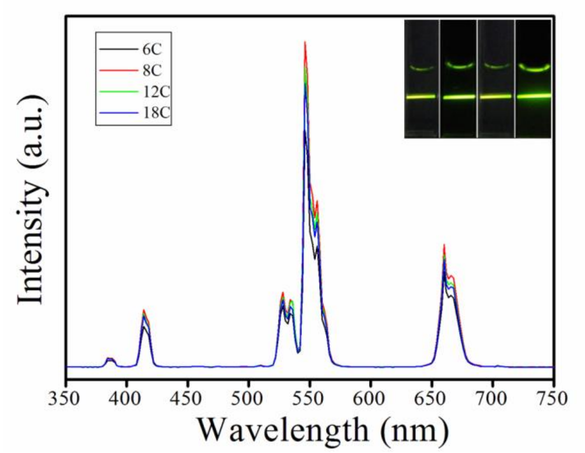

Supplementary Figure S6. The up-conversion emission spectra of 6C, 8C, 12C, 18C silane modified UCNPs under 980nm excitation are similar. The inset shows the digital photo of UCNPs@silane dispersed in water under 980nm laser excitation.

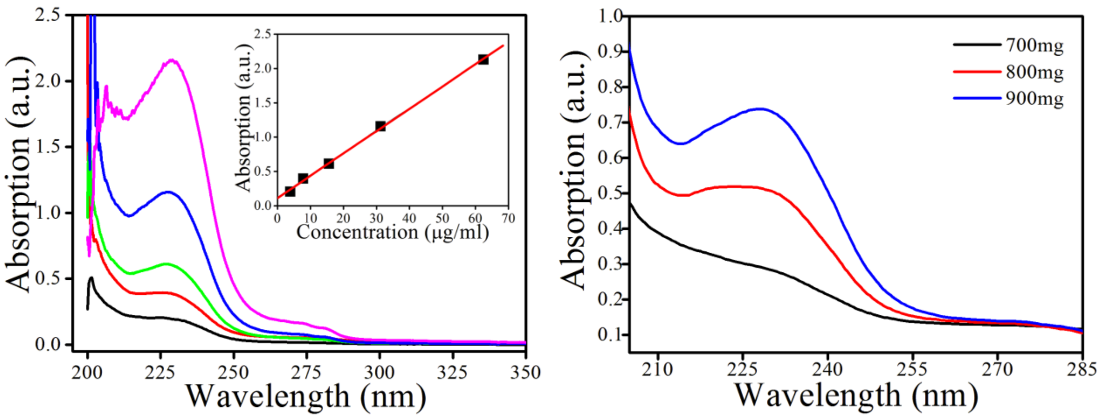

Supplementary Figure S7. (a) The absorption spectra of PTX in different concentration are recorded. The inset is the standard curve: the absorption intensity increases accompanied with the increase of PTX. (b) The absorption spectra of the precipitation's solution are given. As the ratio of PTX to $NaYF_4:Yb^{3+}$, Er^{3+} UCNPs was improved to 0.7:5, 0.8:5, and 0.9:5, there would be white precipitation of PTX appearing. After being dried, the precipitation was resolved in ethanol (5ml). Compared with the standard curve, the loading amount of PTX can be calculated according to the value at 227nm from the absorption spectra.

**Ratio**	**0**	**0.5:5**	**0.6:5**	**0.7:5**	**0.8:5**	**0.9:5**
**EE**	**0**	**100%**	**100%**	**96.1%**	**92.3%**	**87.7%**
**DLE**	**0**	**3.85%**	**4.62%**	**5.1%**	**5.58%**	**5.94%**
**PDI**	**0.203**	**0.26**	**0.193**	**0.343**	**0.252**	**0.285**

Supplementary Table S1. The relationship between encapsulation efficiency (EE), drug loading efficiency (DLE), PDI (Polydispersity index) and the ratio of PTX to $NaYF_4:Yb^{3+}$, Er^{3+} UCNPs.

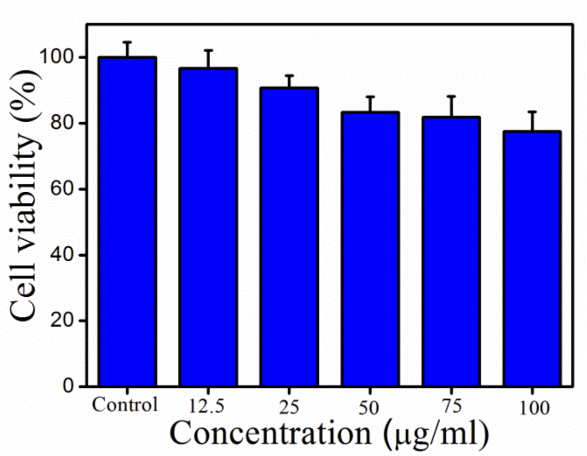

Supplementary Figure S8. Relative cell viability after treatment with different concentrations of UCNPs@PtOEP@silane for 48 h. The experiments were repeated three times, all with similar results. The data are presented as the mean ± SD (for each group, n = 3)

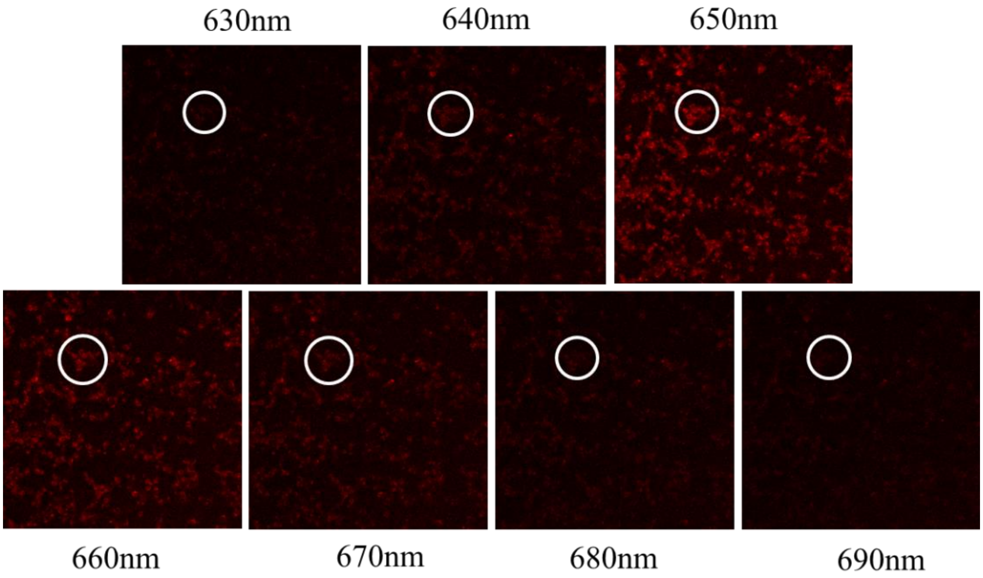

Supplementary Figure S9. Confocal fluorescence images were caught as the detection wavelength changes (at 630, 640, 650, 660, 670, 680 and 690nm). The emission intensity of UCNPs@PtOEP@silane in the ROI was monitored. The step was set as 10nm, and bandwidth was set as 15nm. It clearly shows that the image at 650nm is brightest, meaning that PtOEP obtains maxima at 650nm.
